# MdSCL8 as a Negative Regulator Participates in ALA-Induced *FLS1* to Promote Flavonol Accumulation in Apples

**DOI:** 10.3390/ijms23042033

**Published:** 2022-02-12

**Authors:** Haiwen Zhang, Huihui Tao, Hao Yang, Liuzi Zhang, Guizhi Feng, Yuyan An, Liangju Wang

**Affiliations:** College of Horticulture, Nanjing Agricultural University, Nanjing 210095, China; 2019104008@njau.edu.cn (H.Z.); 18879316621@163.com (H.T.); 2021204010@stu.njau.edu.cn (H.Y.); 2020204011@stu.njau.edu.cn (L.Z.); fgz@njau.edu.cn (G.F.)

**Keywords:** ALA, apple, flavonol, *MdFLS1*, MdSCL8

## Abstract

Apples (*Malus domestica*) are rich in flavonols, and 5-aminolevulinic acid (ALA) plays an important role in the regulation of plant flavonoid metabolism. To date, the underlying mechanism of ALA promoting flavonol accumulation is unclear. Flavonol synthase (FLS) is a key enzyme in flavonol biosynthesis. In this study, we found that ALA could enhance the promoter activity of *MdFLS1* in the ‘Fuji’ apple and improve its expression. With *MdFLS1* as bait, we screened a novel transcription factor MdSCL8 by the Yeast One-Hybrid (Y1H) system from the apple cDNA library which we previously constructed. Using luciferase reporter assay and transient GUS activity assay, we verified that MdSCL8 inhibits the activity of *MdFLS1* promoter and hinders *MdFLS1* expression, thus reducing flavonol accumulation in apple. ALA significantly inhibited *MdSCL8* expression. Therefore, ALA promoted the expression of *MdFLS1* and the consequent flavonol accumulation probably by down-regulating MdSCL8. We also found that ALA significantly enhanced the gene expression of MdMYB22 and MdHY5, two positive regulators of *MdFLS*. We further demonstrated that MdMYB22 interacts with MdHY5, but neither of them interacts with MdSCL8. Taken together, our data suggest MdSCL8 as a novel regulator of *MdFLS1* and provide important insights into mechanisms of ALA-induced flavonol accumulation in apples.

## 1. Introduction

Flavonol, a key class of secondary metabolites, plays important roles in preventing and treating human tumors, preventing cardiovascular diseases, lowering blood sugar and blood lipid, and resisting oxidation, viruses and allergies [1]. For plants, flavonol participates in auxin transportation [2], root growth [3], and the adaptation of plants to the ever-changing environment [4]. The apple (*Malus domestica*) is cultivated and consumed worldwide. Apples are rich in flavonol, the content of which accounts for 9.3% to 70.6% of total polyphenols [5]. Understanding flavonol biosynthesis regulation is crucial to improv fruit quality, increase nutritional value, and enhance environmental adaptability of apples.

The biosynthesis of plant flavonoids begins with phenylalanine. It is catalyzed by enzymes such as Phenylalanine ammonia-lyase (PAL), Chalcone synthase (CHS), Chalcone isomerase (CHI), etc. to form dihydroflavonol. The flavonol synthase (FLS) and dihydroflavonol-4-reductase (DFR) compete for dihydroflavonol to form flavonol and anthocyanin, respectively. Therefore, FLS plays a key role in the biosynthesis of flavonol [6]. The expression level of *FLS* was proved to directly affect the content of flavonols in plants, such as petunia [7], citrus [8], grape [9], peach [10], blueberry [11], and apple [12]. Usually, the expression level of *FLS* is positively correlated with flavonol content. 

Transcription factors regulating the accumulation of flavonols were continuously identified. MYB transcription factors were the most frequently reported, such as MYB111 and MYB12 in *Arabidopsis thaliana* [13], CsMYB60 in cucumbers [14], VvMYBF1 in grapes [15], PbMYB12b in pears [16], and MdMYB22 [17] and MdMYB8 [18] in apples. They were reported to positively regulate flavonol biosynthesis. As an exception, FaMYB1 in strawberries negatively regulates gene expression and enzyme activity at a late stage of flavonoid biosynthesis [19]. These results indicated that the synthesis of flavonol was strictly regulated by positive and negative transcription factors. Excepting MYBs, other transcription factors were increasingly proved to participate in flavonol regulation. For example, in apples, except for MdMYB22 [17] and MdMYB8 [18], MdHY5 [20] and MdNAC9 [21] were also reported to enhance the activity of the *FLS* promoter and promote the flavonol accumulation. These results provide a theoretical basis for the regulatory mechanism of fruit flavonol synthesis.

5-Aminolevulinic acid (ALA), a natural and environmentally friendly multifunctional plant growth regulator [22,23], has been extensively verified for its efficacy in improving plant resistance to abiotic stress [24,25], leaf photosynthetic performance [26], and anthocyanin accumulation [27,28]. Importantly, ALA has been proven to significantly promote the accumulation of flavonol in guard cells of *Arabidopsis thaliana* [29], ‘Fuji’ apple fruits [30], and apple calli [31]. Using ALA is undoubtedly a safe and effective method to improve the content of phytoflavonol. ALA promoted the expression of structural genes related to the flavonoid pathway to influence flavonol accumulation in plants [27,31,32,33]. In addition, ALA can participate in the regulation of gene expression of transcription factors to promote the accumulation of plant anthocyanin [28,30,33]. So far, ALA has been proven to enhance flavonol accumulation in plants; however, the mechanism still remains largely unknown.

MdFLS1 is the key enzyme in apple flavonol biosynthesis [6]. In this study, we found that the activity of *proMdFLS1* and expression level of *MdFLS1* was enhanced by ALA. A novel transcription factor, MdSCL8 as a negative regulator, and two known positive regulators of *MdFLS1*, MdMYB22 and MdHY5, were screened and verified to participate in ALA-promoted expression of *MdFLS1* and flavonol accumulation. This study aimed to provide a theoretical basis for the application of ALA in improving the nutritional and health care quality of fruits. The results will be helpful to enrich the regulatory theory of flavonol biosynthesis.

## 2. Results

### 2.1. ALA Promotes Flavonol Accumulation and Enhances MdFLS1 Expression

**Figure 1:**
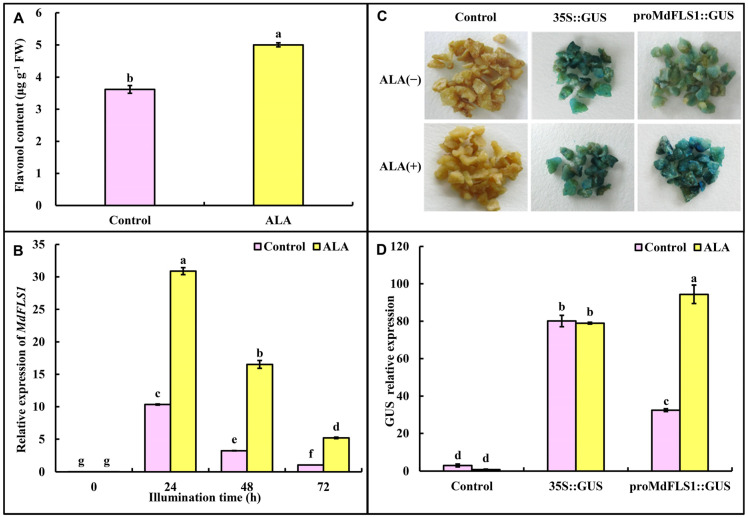
Effect of ALA treatment on flavonol content, *MdFLS1* expression and promoter activity in apple calli. (**A**) Flavonol content of calli under light for 48 h. (**B**) The effect of ALA treatment on *MdFLS1* expression in apple calli. (**C**) GUS staining assays in apple calli. (**D**) Relative expression of *GUS* in apple calli transfected with *35S::GUS* or *proMdFLS1::GUS*. Three biological replicates were performed for each experiment. Error bars represent standard errors. Different lowercase letters in (**A**,**B**,**D**) indicate significant differences among treatments at *p* ≤ 0.05.

The significantly higher level of flavonol content in apple calli treated by 50 mg L^−1^ ALA (Figure 1A) confirmed the promotive effect of ALA on flavonol accumulation. Since MdFLS1 is the key biosynthetic enzyme in apple flavonol biosynthesis, we investigated the effect of ALA on *MdFLS1* expression to explore how ALA regulates flavonol accumulation. We found that ALA significantly up-regulated *MdFLS1* expression (by about 2.5–20 times, Figure 1B). To reveal the regulatory effect of ALA on *MdFLS1*, the promoter of *MdFLS1* upstream of the β-glucuronidase reporter gene (*GUS*) was constructed. Transient expression assays in apple calli showed that the *proMdFLS1*::GUS transgenic staining was significantly deepened after ALA treatment, and GUS enzyme activity was significantly increased (Figure 1C,D). These results indicate that ALA-improved flavonol accumulation is closely related to the higher *proMdFLS1* activity and *MdFLS1* expression induced by ALA.

### 2.2. MdSCL8 Binds to MdFLS1 Promoter and Inhibits Its Activity

**Figure 2:**
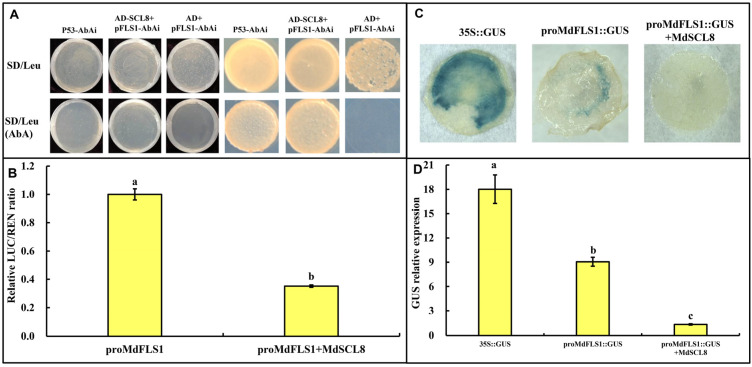
Interaction of MdSCL8 with the promoter of *MdFLS1*. (**A**) Yeast one-hybrid assays identified interaction of MdSCL8 with *proMdFLS1*. Left: Yeast transformation system coating plate. Right: yeast-positive monoclonal spot plate. pGADT7-53 plasmid was transferred into p53-AbAi yeast bait as the positive control, and empty pGADT7-AD was transferred into *proMdFLS1*-AbAi yeast bait as the negative control. SD/-Leu and SD/-Leu (AbA) indicate leucine-deficient boards without and with 400 ng mL^−1^ Aureobasidin A (AbA), respectively. (**B**) The effects of MdSCL8 on the promoter activity of *MdFLS1* with the luciferase reporter assay. (**C**) GUS staining assays in tobacco leaves of *35S::GUS*, *proMdFLS1::GUS* transfection and *proMdFLS::GUS+MdSCL8* co-transfection. (**D**) Relative expression of GUS in tobacco leaves transfected with *35S::GUS*, *proMdFLS1::GUS* or *proMdFLS::GUS*+*MdSCL8*. Three biological replicates were performed for each experiment. Error bars represent standard errors. Different lowercase letters in (**B**,**D**) indicate significant differences at *p* ≤ 0.05.

To excavate the specific regulators in ALA-induced flavonol accumulation, we constructed bait yeast [*proMdFLS1*-AbAi] to screen the cDNA library of ALA-treated apple calli (Figure S1). The results showed that the proteins screened were mainly involved in the regulation of photosynthesis, carbon and glucose metabolism, response to oxidative/osmotic stress, hormone signal transduction, lignin biosynthesis, fruit maturation and development, and some proteins with unknown functions (Table 1). Among them was transcription factor MdSCL8, the expression of whose homologous gene in strawberries was previously reported to be related to synthesis of flavonoid compounds [34,35]. Yeast one-hybrid assays confirmed that MdSCL8 interacts with *proMdFLS1*. In the Y1H assay, the positive control (p53-AbAi) grew normally on SD/-Leu or SD/-Leu (AbA) medium, while the negative control (AD+p*FLS1*-AbAi) did not. The transformation of the PGADT7-MdSCL8 plasmid into p*MdFLS1*-AbAi yeast competence resulted in normal growth on either SD/-Leu or SD/-Leu (AbA) media, indicating that MdSCL8 activates the expression of the reporter gene in p*MdFLS1*-AbAi to allow normal growth on AbA-containing plates (Figure 2A). To investigate whether and how MdSCL8 regulates *MdFLS1*, we determined the promoter activity of *MdFLS1* by dual-luciferase assays. The results showed that the promoter activity of *MdFLS1* was strongly inhibited by MdSCL8 (Figure 2B). Transient expression assays in tobacco leaves further showed that when *MdSCL8* and *proMdFLS1* were co-transferred, the GUS activity was only 15% of that in the single-transferred leaves of *proMdFLS1* (Figure 2C,D). These results indicate that MdSCL8 negatively regulates the activity of *MdFLS1* promoter.

### 2.3. Identification and Molecular Characterization of MdSCL8

**Figure 3:**
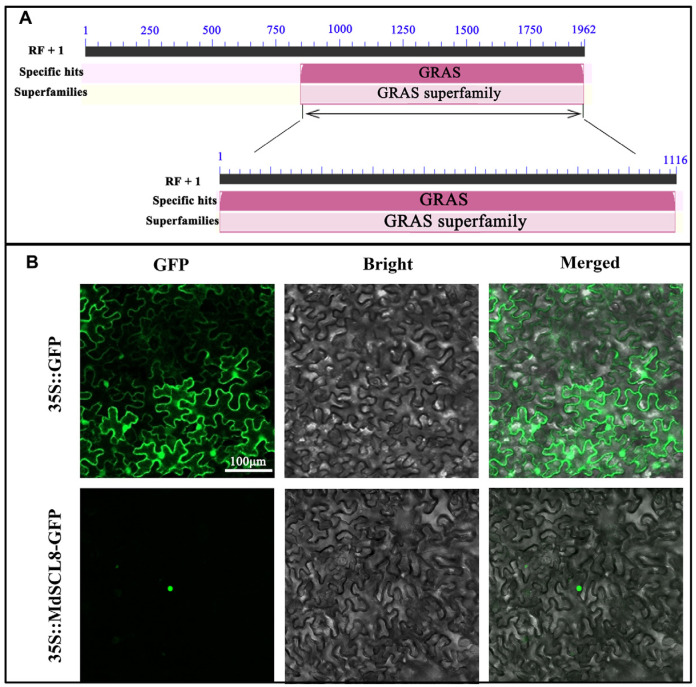
Analysis of conserved domain and subcellular localization of MdSCL8 in tobacco leaves. (**A**) The functional domain predication of MdSCL8. (**B**) MdSCL8 subcellular localization. GFP fluorescence signal was observed by confocal microscope at 488 nm. GFP, Bright, and Merged represent green fluorescence, bright field, and merged field, respectively. The scale is 100 µm.

The full-length coding sequence (CDS) of *MdSCL8* was cloned from a ‘Fuji’ apple. The length of the protein encoded by *MdSCL8* contains 653 amino acids. The protein MdSCL8 is weakly acidic, and *MdSCL8* is located on chromosome 7 with a molecular weight of 71.38 kDa and an isoelectric point of 6.42. The extinction coefficients are 0.545 and 0.538, and the stability coefficient is 51.56. In amino acid composition, Serine (Ser) and Alanine (Ala) are the most abundant, accounting for 11.3% and 7.8%, respectively. Total mean hydrophilicity index is −0.506, suggesting it is a hydrophilic protein. In addition, conserved domains prediction reveals a typical conservative GRAS domain at the C-terminal of MdSCL8 (Figure 3A). In the tobacco leaves which were injected with a vector of 35S:MdSCL8-GFP, we found that the GFP signal of MdSCL8 was located in the nucleus, while the GFP signal of 35S::GFP was detected throughout the cells (Figure 3B). 

### 2.4. MdSCL8 Negatively Regulates Flavonol Accumulation and ALA Suppresses This Process

**Figure 4:**
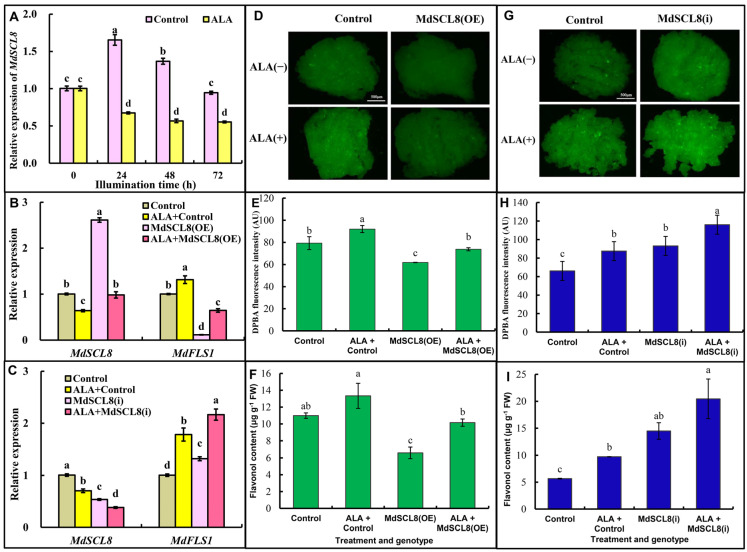
Functional characterization of *MdSCL8*. (**A**) *MdSCL8* expression in apple calli with ALA treatment. (**B**) Gene expression levels in *MdSCL8* overexpressing transgenic calli *(OE)*. (**C**) Gene expression levels in *MdSCL8*-interfering calli *(i)*. (**D**) DPBA staining of *MdSCL8(OE)* apple calli. The scale is 500 µm. (**E**) DPBA fluorescence intensity in *MdSCL8(OE)* apple calli. (**F**) The flavonol content of *MdSCL8(OE)* transgenic calli determined by UPLC. (**G**) DPBA staining of *MdSCL8(i)* apple calli. The scale is 500 µm. (**H**) DPBA fluorescence intensity in *MdSCL8(i)* transgenic calli. (**I**) The flavonol content of *MdSCL8(i)* transgenic calli determined by UPLC. Control of (**B**,**D**–**F**) was corresponding to PBI121 vector, Control of (**C**,**G**–**I**) was corresponding to PHG2 vector. Three biological replicates were performed for each experiment. Error bars represent standard errors. The same lowercase letters in (**A**–**C**,**E**,**F**,**H**,**I**) indicate no significant differences at *p* ≤ 0.05.

To verify the role of MdSCL8 in the accumulation of flavonol, apple calli were treated with ALA under light for 0 h, 24 h, 48 h and 72 h, respectively. As shown in Figure 4A, the expression of *MdSCL8* was up-regulated after illumination, which was significantly inhibited by ALA treatment. We further established an overexpressing *MdSCL8* transgenic apple callus and an RNA interference transgenic line, *MdSCL8(i)*. Compared with their corresponding control, the expression of *MdSCL8* was increased by 150% in *MdSCL8(OE)* and decreased by 50% in *MdSCL8(i)* (Figure 4B,C). The expression of *MdFLS1* was reduced by 90% in *MdSCL8 (OE)* and increased by 130% in *MdSCL8 (i)*, compared with their control. Consequently, the flavonol content in *MdSCL8(OE)* transgenic calli was significantly reduced, and that in *MdSCL8(i)* was significantly increased (Figure 4D–I). These results suggest a negative role of MdSCL8 in apple flavonol accumulation. ALA treatment decreased *MdSCL8* expression by 36% in the control group, and by 62% in the *MdSCL8(OE)* strains. Meanwhile, *MdFLS1* increased by 30% and 450% in the control group and *MdSCL8(OE)* strains, respectively (Figure 4B). This result implies that ALA can strengthen its inhibitory effect on *MdSCL8* in *MdSCL8(OE)* line to release more *FLS1*. Consistently, ALA increased relatively more flavonol accumulation in *MdSCL8(OE)* line (54%) than in control (21%) (Figure 4F). In the *MdSCL8* interfering experiment, ALA inhibited *MdSCL8* expression and increased *MdFLS1* expression in both the control group and *MdSCL8(i)* line (Figure 4C), thus significantly enhancing their flavonol accumulation (Figure 4H,I). These results indicate that MdSCL8 plays a negative regulatory role in flavonol biosynthesis, and may participate in ALA-regulated flavonol accumulation.

### 2.5. Regulatory Effects of MdHY5 and MdMYB22 in ALA-Induced Flavonol Accumulation

**Figure 5:**
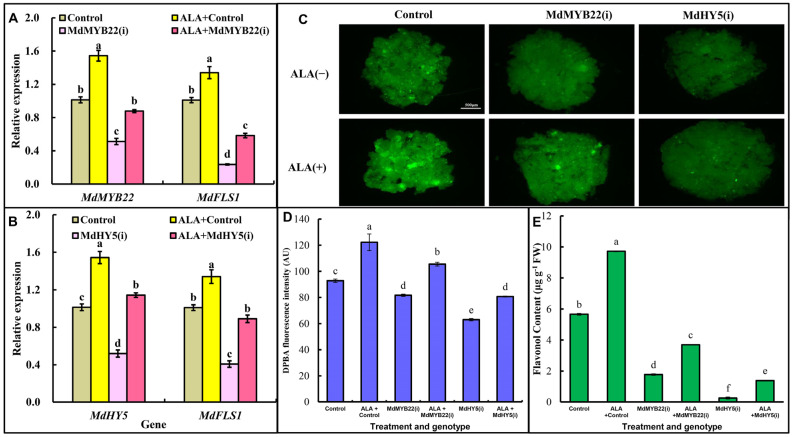
Effect of ALA on the flavonol content as well as the gene expression in *MdMYB22(i)* and *MdHY5(i)* transgenic calli. (**A**) Gene expression levels in *MdMYB22* interfering transgenic calli. (**B**) Gene expression levels in *MdHY5* interfering transgenic calli. (**C**) DPBA staining of *MdMYB22(i)*, *MdHY5(i)* apple calli. The scale is 500 µm. (**D**) DPBA fluorescence intensity of *MdMYB22(i)* and *MdHY5(i)* apple calli. (**E**) The flavonol content of *MdHY5(i)* and *MdMYB22(i)* transgenic calli determined by UPLC. Three biological replicates were performed for each experiment. Error bars represent standard errors. Different lowercase letters in (**A**,**B**,**D**,**E**) indicate significant differences at *p* ≤ 0.05.

MdHY5 [20], MdMYB8 [18], MdMYB22 [17] and MdNAC9 [21] are positive regulators in apple flavonol accumulation. Hence, we verified the relationship between ALA and these regulatory factors. The expression levels of *MdHY5* and *MdMYB22* were up-regulated by ALA (Figure S2), while that of *MdNAC9* and *MdMYB8* were not significantly affected (data not shown). This result indicates that ALA may stimulate flavonol accumulation by improving the expression of *MdMYB22* and *MdHY5*. Next, *MdMYB22* and *MdHY5* were RNA-interfered in apple calli to do further analysis. The expression levels of *MdMYB22* and *MdHY5* were both reduced by 50% in interference lines, and *MdFLS1* expression was reduced by 77% and 59%, respectively. ALA increased the expression of *MdMYB22* and *MdHY5* both by 52% in the control group, and by 70% and 120%, respectively, in the corresponding interfering strains. *MdFLS1* was increased by ALA by 33% in the control group and by 150% and 120%in the *MdMYB22(i)* and *MdHY5(i)* strains, respectively (Figure 5A,B). Flavonol content significantly decreased in the two interfering transgenic lines compared to the control (Figure 5C–E). ALA increased the flavonol content by 72% in the control group, and by 108% and 450% in the *MdMYB22(i)* and *MdHY5(i)* lines, respectively (Figure 5E). These results indicate that ALA displays a compensating effect on flavonol accumulation when the MdMYB22 or MdHY5 pathway was suppressed. In other words, *MdMYB22* and *MdHY5* may be involved in the accumulation of flavonol induced by ALA, and some other pathways also exist.

### 2.6. MdMYB22 and MdHY5 Interact, and Neither of Them Interacts with MdSCL8

**Figure 6:**
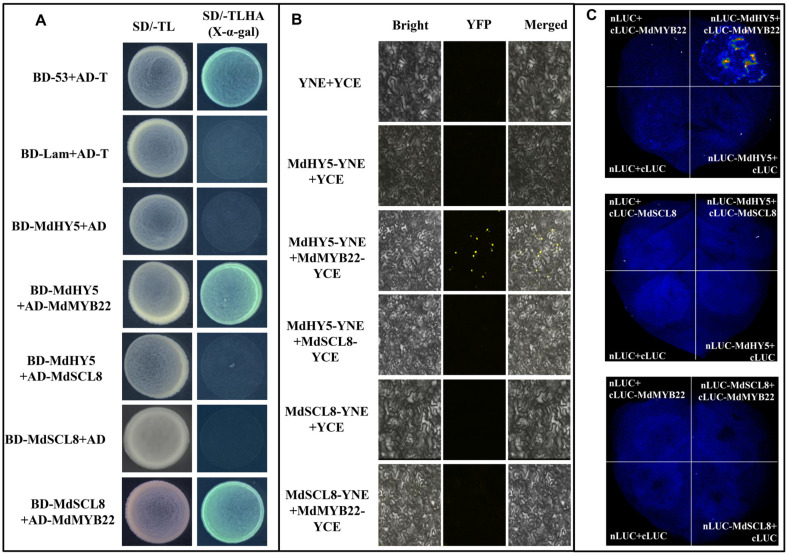
Analyses of the interactions between MdHY5, MdMYB22, and MdSCL8. (**A**) Yeast two-hybrid analysis. SD/-TL is SD/–Leu/–Trp solid medium, SD/-TLHA (X-α-gal) is SD/-Leu/-Trp/-His/-Ade/X-α-gal solid medium. (**B**) BiFC assay analysis of the interactions in tobacco leaves. YFP fluorescence signal was observed by confocal microscope at 510 nm. Bright, YFP, and Merged correspond to bright field, yellow fluorescence, and merged field, respectively. (**C**) Luciferase assay analysis in vivo imaging system. Three biological replicates were performed for each experiment.

Since MdSCL8, MdMYB22 and MdHY5 all participate in ALA-induced flavonol accumulation, it is interesting to investigate whether they interact with each other. In the Yeast two-hybrid (Y2H) assay, the negative control will not grow on SD/-Leu/-Trp/-His/-Ade (X-α-gal) medium, while the positive control and the yeast transformed with the interacting proteins grew normally and turned blue. When the two proteins interacted, the AD and BD domains to which they were connected were close in space, thus activating the expression of the reporter gene. The Y2H results showed that BD-MdHY5 interacted with AD-MdMYB22, and BD-MdMSCL8 interacted with AD-MdMYB22, but MdHY5 did not interact with MdMSCL8 (Figure 6A). To confirm this result, we performed bimolecular fluorescence complementarity assay (BIFC). Results showed that MdHY5-YNE and MdMYB22-YCE interacted with each other, consistent with the Y2H result (Figure 6B). However, in contrast to the Y2H results, there was no fluorescence between MdSCL8-YNE and MdMYB22-YCE. To further validate the interaction, we performed a double luciferase in vivo imaging experiment in tobacco leaves. Results showed that the fluorescence occurred only in the regions of co-transNLUC-MdHY5 and cLUC-MdMYB22, suggesting that only MdHY5 interacts with MdMYB22 (Figure 6C). These results indicate that there is an interaction between MdMYB22 and MdHY5, without the involvement of MdSCL8. 

## 3. Discussion

### 3.1. ALA Positively Regulates MdFLS1

Flavonol is an important class of polyphenols which plays a crucial role in the oxidation resistance and prevention of oxidative stress-related diseases, such as cardiovascular disease [36] and cancer health protection [37]. Apples are one of the most important fruits that consumers often intake as one source of flavonol [38]. Therefore, increasing the content of flavonol is of great significance for improving the nutrition and health benefits of apple fruits. It was reported that the flavonol content was affected by many factors, such as light [39], temperature [40], plant growth regulator [41], and cultivation factors [42]. ALA, a natural and non-toxic plant growth regulator [23], has attracted the attention of many scholars due to its important roles in improving plant resistance [24,25] and fruit quality [27]. ALA promoting flavonol accumulation was first proved in guard cells of apple [43] and Arabidopsis [29], which scavenged reactive oxygen species and induced stomatal opening. In apple fruits [30] and calli [31], ALA was found to promote the content of quercetin and kaempferol. In this study, we also found that ALA promoted the accumulation of flavonol in the apple calli using both DPBA and UPLC methods (Figure 1A). Therefore, ALA-promoted phytoflavonol accumulation is universal, which provides a safer and more effective way to improve phytoflavonol accumulation.

FLS is a key enzyme in the branch of flavonol biosynthesis. It plays a key role in the biosynthesis of flavonol [6]. *FLS* genes have been cloned and their functions have been verified in a variety of plants, such as *Arabidopsis thaliana* [44], tobacco [45], tartary buckwheat [46], and corn [47]. The expression of *FLS* is affected by a variety of external environments, and it is positively correlated with the content of flavonol. There have been many reports that plant growth regulators participated in the regulation of flavonol biosynthesis by affecting the expression of *FLS*. In *Ginkgo biloba*, *GbFLS1* expression was induced by abscisic acid (ABA), ethylene (ETH), and salicylic acid (SA), with a concomitant increase in flavonol content [48]. SA induced up-regulation of *VcFLS* expression in *Vaccinium myrtillus* [11]. ABA induced up-regulation of *MdFLS1* expression in plantlets of ‘Gala’ apple in vitro [12]. Treatments of ‘Fuji’ apples with 24-epibrassinolide and ALA promoted the accumulation of quercetin and kaempferol [31]. ALA has been shown to promote flavonol accumulation and up-regulate *MdFLS* [30]. In the present study, we further found that ALA significantly increased the activity of *MdFLS1* promoter, which might be the direct reason for ALA increasing the expression abundance of *MdFLS1* (Figure 1C,D). These results indicate that ALA positively regulated *MdFLS1* expression, and ALA-improved flavonol accumulation is probably achieved by regulating *MdFLS1* expression.

### 3.2. ALA-Inhibited MdSCL8 Negatively Regulates MdFLS1 and Flavonol Accumulation in Apples

Flavonol synthesis is regulated by various transcription factors, such as HY5 [49] and MYB22 [17]. In this study, the promoter sequence of *MdFLS1* was used as a bait to screen the functional proteins that may interact with the promoter (Figure S1, Table 1). Interestingly, MdSCL8, a new transcript factor, was screened first, which has never been mentioned in the regulation of *MdFLS1* expression and accumulation of flavonoids before.

SCL8 belongs to the GRAS family, which plays an important role in regulating transcription and plant signal transduction pathway [50,51]. The apple GRAS gene family contains 127 genes, which are divided into eight subtribes according to phylogenetic tree: DELLA, SCL3, SCR, LS, LISCL, PAT1, SHR, and HAM [52]. MdSCL8 belongs to the PAT1 subfamily. The PAT1 subfamily plays a role in light-sensitive signal transduction and response to abiotic stress [53]. The transcription factors in the PAT1 subfamily reported in Arabidopsis are PAT1, SCL21, and SCL13. PAT1 and SCL21 are positive regulatory factors for the signal transduction of phytochrome A pathway [53,54]. In this study, MdSCL8 was proved to interact with *MdFLS1,* and overexpression of MdSCL8 significantly inhibited the activity of *FLS1* promoter (Figure 2). Genetic data further suggest that MdSCL8 is a negative regulator of *MdFLS1* expression and flavonol accumulation in apples, while ALA negatively regulates *MdSCL8* expression (Figure 4). Interestingly, when *MdSCL8* was overexpressed, ALA strengthened its inhibitory effect on *MdSCL8* (Figure 4). This is the first report that a GRAS transcription factor is involved in the regulation of fruit flavonol biosynthesis.

### 3.3. MdHY5 and MdMYB22 Are Involved in ALA-Induced Flavonol Accumulation in Apples

Many transcription factors have been reported to be involved in the regulation of flavonol accumulation, among which the MYB family is the most important. VvMYBF1 in grapes [15], MdMYB22 [17] and MdMYB8 [18] in apples, and PpMYB17 [55] and PbMYB12b [16] in pears, are all suggested to bind and activate *FLS* promoter, leading to the accumulation of flavonols. The bZIP family, such as AtHY5 in Arabidopsis [49], VvbZIPC22 in grapes [56], CsHY5 in tea [57], and MdHY5 in apples [20], have also been suggested to indirectly regulate *FLS* by regulating other transcription factors, and promote the accumulation of flavonols. In addition, MdNAC9 in apple has also been shown to promote flavonol accumulation [21]. Here, we found that the expression levels of *MdMYB22* and *MdHY5* were significantly increased in ALA-treated calli (Figure S2). *MdMYB22* and *MdHY5* still respond to ALA treatment after interference (Figure 5). ALA increased the expression levels of *MdMYB22* and *MdHY5* in the *MdMYB22(i)* and *MdHY5(i)* strains much more obviously than in the control group, which in turn increased the expression of *MdFLS1* and improved the content of flavonol. This result is similar to the effect of ALA on *MdSCL8.* It seems that multiple pathways are involved in ALA-induced flavonol accumulation, and other pathways will compensate when one pathway is suppressed. How these pathways work synergistically is worthy to be further studied. HY5 has been considered as the center of a transcriptional network hub, which promotes flavonol biosynthesis through combinatorial action of MYB and bHLH transcription factors [58]. In this study, we proved the interaction of MdMYB22 with MdHY5, but neither of them interacted with the negative regulator MdSCL8 (Figure 6). Our data suggest three pathways for ALA-induced flavonol accumulation, providing new insights to the regulation of flavonol biosynthesis, but also indicate that some other pathways are involved in this process. We will continue to explore the molecular mechanism of ALA-induced accumulation of flavonol in apples.

## 4. Materials and Methods

### 4.1. Experimental Materials and Treatments

The calli of apples were cultured in the Fruit Physiology and Ecology Laboratory of College of Horticulture, Nanjing Agricultural University according to the method described by An et al. [59]. Apple calli were treated by ALA according to previous methods in our lab [30]. The calli of ‘Fuji’ and ‘Orin’ apples were treated with 50 mg L^−1^ ALA or deionized water (Control) for 3 h in darkness and then cultured in solid MS medium under light of 100 µmol m^−2^s^−1^ photon flux density at 22 °C. Samples were collected after 0 h, 24 h, 48 h, and 72 h of illumination.

### 4.2. The Content of Flavonol in Apples Was Determined by UPLC

Quercetin-3-galactoside, rutin, quercetin, and kaempferol from Yuanye Company (Shanghai, China) were used as standard samples. The standard samples were retained in methanol at −40 °C with a concentration gradient of 0.25 mg L^−1^, 0.5 mg L^−1^, 1 mg L^−1^, 1.5 mg L^−1^, and 2 mg L^−1^. 0.5 g apple calli were weighed, ground with liquid nitrogen, and extracted with 100 Hz ultrasound for 30 min after being immersed in 80% methanol (80:20, MeOH/H_2_O) overnight in a 4 °C refrigerator. Samples were then centrifuged at 12,000 r min^−1^ for 20 min. The supernatants were collected and filtered through a 0.45 µm filter before utilization. Samples were measured by an UltiMate 3000 UPLC (Thermo, Waltham, MA, USA) equipped with a 2.1 × 100 mm Column. Each injection volume was 2 µL. The mobile phase was composed of (A) 0.1% acetic acid water and (B) acetonitrile at a flow rate of 0.35 mL min^−1^. The gradient elution was performed as follows: 0 min, (B) 5%; 2.8 min, (B) 55%; 3.5 min, (B) 60%; 4 min, (B) 5%; and 6 min, (B) 5%. The UV absorbance peaks were monitored at 288 nm. 

### 4.3. DNA Extraction, RNA Extraction, and qRT-PCR

Total DNA/RNA of apple calli was isolated using the plant total DNA/RNA extraction kit (Tiangen, Beijing, China). First-strand cDNA was synthesized using a RevertAid^TM^ First Strand cDNA Synthesis Kit (Transgen, Beijing, China). The primers (Table S1) were designed with Primer Premier software (Premier, Canada) and synthesized by Generay Biotechnology (Shanghai, China). *MdUBQ* was used as the internal control. Taking cDNA as template, the qRT-PCR reactions were performed on a Quantitative real-time PCR instrument (ABI, Los Angeles, CA, USA) according to the instructions of Chamq SYBR qPCR master mix (Vazyme, Nanjing, China), and 2^−^^ΔΔCT^ method was used for calculation [60].

### 4.4. Construction and Infection of MdFLS1 Promoter Vector

*MdFLS1*(MDP0000260404) of ‘Fuji’ apple was obtained in Genome Database for Rosaceae (GDR). Taking ‘Fuji’ apple DNA as template, the promoter of *MdFLS1* was cloned by 2 × Phanta Max Master Mix (Vazyme, Nanjing, China). The PBI121 vector was linearized with *Flycut^®^* mix (Transgen, Beijing, China) and the *35S* promoter was excised for recombinant transformation.

*Agrobacterium tumefaciens* GV3101 with recombinant plasmid was propagated to 0.5 OD_600 nm_, and the cells were re-suspended by using suspension solution (100 mM MES, 100 mM MgCl_2_, 150 mM AS) to 0.5 OD_600 nm_. Apple calli were infected by the above suspension solution for 15-30 min and then the calli were transferred to solid MS medium containing 50 mg L^−1^ ALA in darkness at 22 °C for 48 h. After another 48 h of light culture, samples were collected for qualitative and quantitative GUS experiment. 

### 4.5. Bioinformatics Analysis

The amino acid sequence of MdSCL8 was downloaded from the NCBI database; meanwhile, the chromosomal location, CDS sequence, and the number of exons and introns were found in NCBI. The physicochemical properties of protein, such as the number of amino acids, isoelectric point, molecular weight, and other information on protein were analyzed by ExPASY online. The hydrophobicity of protein was analyzed using the online tool Protscale. The NCBI conserved domains tool was used for protein conservative functional domain prediction. Amino acid sequences were aligned using DNAMAN software (Lynnon Biosoft, San Ramon, CA, USA). 

### 4.6. Yeast One-Hybrid Assay

The expression vector of *proMdFLS1*-AbAi was constructed using *proMdFLS1* as the bait sequence. The CDS region of *MdSCL8* was cloned into pGADT7 vector to form *PGADT7-MdSCL8*. The *proMdFLS1*-AbAi plasmid linearized by BstBI restriction endonuclease (NEB, Beijing, China) was transformed into yeast competence to obtain bait yeast [*proMdFLS1*-AbAi]. The library screening experiment was conducted according to the yeast one-hybrid instruction of Shanghai OE Biotech Co., Ltd. (Shanghai, China). The screened genes were subjected to yeast colony PCR, sequencing analysis, and point-to-point verification.

### 4.7. MdSCL8, MdHY5, and MdMYB22 Cloning and Transformation into Apple Calli

For the construction of the overexpression vector, the CDS region of *MdSCL8* was amplified and connected to the PBI121 vector. For the construction of the interference vector, the conservative region was searched on the SMART online software according to the amino acid sequence information of MdSCL8, MdMYB22, and MdHY5, and 200–400 bp sequence fragments were selected. After gene cloning and recovery, the fragments were recombined onto PHG2 (pHELLSGATE2) vector by using the Gateway BP™ LR Clonase™ Enzyme Mix (Thermo Fisher, Waltham, MA, USA).

Infection was carried out according to the method of Zheng [28]. The recombinant plasmids were inserted into *A. tumefaciens* GV3101 cells and then transformed in apple calli. The calli were then co-cultured on solid MS medium for 48 h at 22 °C in darkness. The calli were treated with 50 mg L^−1^ ALA or deionized water (Control) for 3 h in darkness and then cultured in solid MS medium under light of 100 µmol m^−2^s^−1^ photon flux density at 22 °C for 48 h.

### 4.8. Subcellular Localization

The full-length MdSCL8 CDS was amplified (with the terminator codon removed) and inserted into pCambia1300 vector for recombinant plasmid. The empty pCambia1300 was used as control. The *A. tumefaciens* GV3101 containing the plasmid was injected into the 5-week-old tobacco (*Nicotiana benthamiana*) leaves. The infiltrated tobacco was placed for 2–3 days in dark conditions. The fluorescence signal of tobacco was detected using ultra-high-resolution confocal microscope (Zeiss, Oberkochen, Germany) with an excitation wave length 488 nm.

### 4.9. Luciferase Reporter Assay

The CDS of *MdSCL8* was ligated to pCambia1300 effector, and the *proMdFLS1* was inserted into pGreenII 0800-LUCreporter. Tobacco leaves were used as the materials for transient transfection. The ‘reporter’ was set as the control, and ‘effector’ and ‘reporter’ were uniformly mixed in a proportion and jointly converted to tobacco leaves. Fifty milligram leaves were ground and operated according to the instruction of the double luciferase reporter gene detection kit (Transgen, Beijing, China). LUC and REN activities were then measured on a nite200Pro microplate reader (Tecan, Männedorf, Switzerland). The luciferase activity was expressed by the ratio of LUC/REN. The control group LUC/REN value was set to 1.

### 4.10. Transient GUS Activity Assays

The ORF of *MdSCL8* was cloned into the pCambia1300 effector, and the promoter of *MdFLS1* was inserted into the PBI121 reporter. The ‘effector’ and ‘reporter’ were mixed and injected into 5-week-old tobacco leaves. After reaction at 22 °C for 48 h, the GUS histochemical assays were measured as previously [14]. X-β-D-glucuronide (X-Gluc) was adsorbed 50 μL into 20 mg mL^−1^ DMF and added to 450 μL of buffer (50 mM phosphate buffer pH 7.0, 10 mM EDTA pH = 8.0, 10% Triton X-100, 50 mM potassium ferricyanide, 50 mM potassium ferrocyanide). The treated tobacco leaves were beaten into disk-shaped leaves, and then 500 μL GUS staining solution was added into the disk-shaped leaves at 37 °C for 12 h. The samples were rinsed with 80% alcohol, and the staining of the leaves was observed. GUS activity was measured using a microplate reader according to the instructions of the GUS enzyme activity detection kit (FCNCS, Nanjing, China).

### 4.11. Analysis of DPBA Staining in Transgenic Calli

The flavonol of apple calli was detected by diphenylboric acid 2-amino-ethyl ester (DPBA) staining. DPBA can bind to flavonols such as quercetin and cause fluorescence under laser light [61]. Twenty-five milligram DPBA powder was weighed and prepared into a 0.25% (*w*/*v*) solution, which was then diluted to 0.005% (*v*/*v*) by adding TritonX-100. After being normally illuminated for 48 h, the transgenic apple calli was treated with DPBA in darkness for 30–60 min. Afterwards, the tissue was observed under GFP light of a stereo fluorescence microscope (Leica, Wetzlar, Germany). The fluorescence intensity of DPBA was analyzed by ImageJ software.

### 4.12. Yeast Two-Hybrid Assay

The CDSs of *MdHY5*, *MdMYB22*, and *MdSCL8* were recombined into PGBKT7(BD) and PGADT7(AD) vectors. The positive controls were PGBKT7-53(BD-53) + PGADT7-T(AD-T) co-transduced, while PGBKT7-Lam (BD-Lam) + PGADT7-T(AD-T) were negative controls. BD-MdHY5 + PGADT7(AD) empty vector and BD-MdSCL8 + AD empty vector also served as controls, respectively. The transformation of the BD-MdHY5 and BD-MdSCL8 plasmids into the Y2H yeast competence assay revealed the absence of self-activation. The recombinant plasmids were co-transferred into Y2H gold yeast competent cells (Weidi, Shanghai, China), coated on SD/-Leu/-Trp solid medium, and incubated at 30 °C for 3 days before colonies were transferred to selection medium (SD/-Ade/-His/-Leu/-Trp/X-α-gal).

### 4.13. Bimolecular Fluorescence Complementation Assays

The CDSs of *MdHY5*, *MdSCL8* and *MdMYB22* were cloned into 35S-pSPYNE-YFP(YNE) and 35S-pSPYCE-YFP(YCE) vectors, with YNE+YCE, YNE-MdHY5+YCE, and MdSCL8-YNE+YCE as the control, respectively. The recombinant plasmid was transformed into the competent *A. tumefaciens* GV3101, and the tobacco leaves were co-transferred and treated for 48h in the dark. The YFP fluorescence signal was detected by LSM800 (Zeiss, Baden-Württemberg, Germany) with an excitation wavelength of 510 nm. 

### 4.14. Determination of Luciferase Transient Expression in Tobacco Leaves

*MdHY5*, *MdSCL8*, and *MdMYB22* were constructed into N terminal of pCambia1300-35S-nLUC vector and the C terminal of pCambia1300-35S-cLUC vector, respectively. The recombinant plasmids were transferred into GV3101, infected tobacco leaves, which were soaked for 48 h. One mM firefly substrate D-luciferin (Yeasen, Shanghai, China) was sprayed on the backs of the leaves, and stood for 7 min under dark conditions. The back of the leaf was placed upward on the plant in vivo imaging system (Princeton Instruments, Trenton, NJ, USA) to detect the luminescence.

### 4.15. Data Analysis

All the data have at least three biological repetitions, and SPSS 20.0 software (SPSS Inc., Chicago, IL, USA) was used for statistical analysis. The analysis of variance (ANOVA) and Duncan multiple range test were used to compare the significant differences at *p* ≤ 0.05 level.

## 5. Conclusions

**Figure 7:**
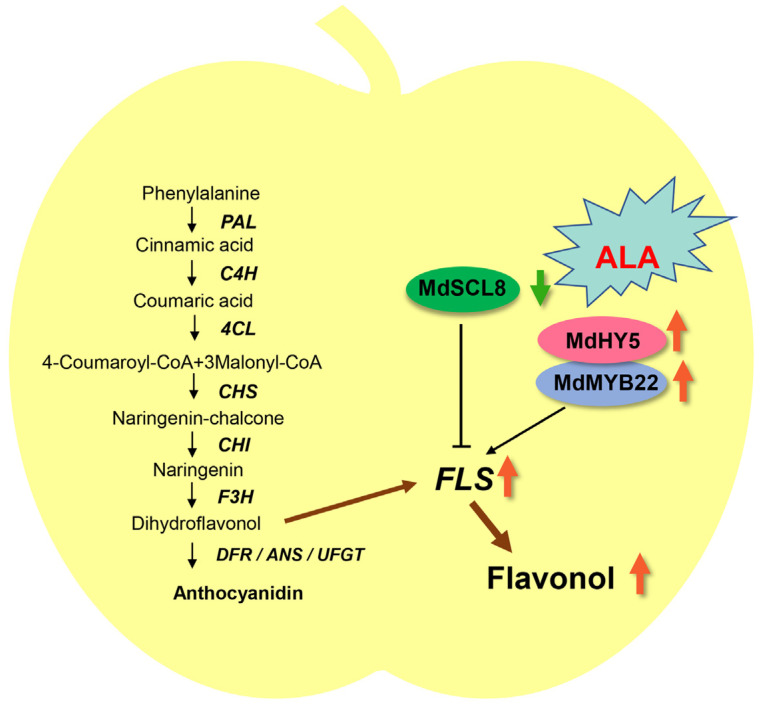
A model proposed for the regulatory network of ALA-mediated flavonol biosynthesis. The line → stands for positive effect and —| stands for negative effect. The orange arrows indicate up-regulation and the green arrows indicate down-regulation of the expression by ALA.

In summary, ALA improves flavonol accumulation in apples by regulating *MdFLS1*. In this process, a novel negative regulator, MdSCL8, which belongs to GRAS family, and two positive regulators, MdMYB22 and MdHY5, were confirmed to play important roles. Based on these results, we proposed a working model for how ALA regulates flavonol biosynthesis (Figure 7). It is the first report to explore the mechanism underlying ALA-induced flavonol accumulation in apples, providing new insights into the regulation of plant flavonol biosynthesis and beneficitting improvement of fruit nutrition for the health of human beings.

## Figures and Tables

**Table 1:** Screened proteins that may interact with the *MdFLS1* promoter.

Number	Accession No.	Gene Description	Similarity
1	NC_041801	1,2-dihydroxy-3-keto-5-methylthiopentene dioxygenase 2	80%
2	NC_041803	probable aquaporin SIP2-1	99%
3	NC_041799	cinnamoyl-CoA reductase 1-like	97%
4	NC_041792	pyridoxal 5’-phosphate synthase subunit PDX1.3	95%
5	NC_041793	NAD(P)H dehydrogenase (quinone) FQR1-like	99%
6	NC_041795	SCL domain class transcription factor (SCL8)	98%
7	NC_041802	zinc finger A20 and AN1 domain-containing stress-associated protein 4	99%
8	NC_041790	proteasome subunit beta type-4-like	98%
9	NC_041800	fructose-bisphosphate aldolase cytoplasmic isozyme-like	99%
10	NC_041789	acyl-CoA-binding domain-containing protein 1-like	99%
11	NC_041799	L-ascorbate peroxidase, cytosolic-like	99%
12	NC_041796	two-component response regulator ORR10-like	96%
13	NC_041800	early responsive to dehydration 15-like	90%
14	NW_007545529	LRR receptor-like serine/threonine-protein kinase GSO1	94%
15	NC_024241	fatty-acid-binding protein 1	92%
16	NC_041801	rRNA-processing protein fcf2-like	99%
17	NC_041792	ubiquitin-conjugating enzyme E2 28	98%
18	NM_001294126	glutathione S-transferase DHAR2-like (DHAR)	99%
19	NC_041794	probable phospholipid hydroperoxide glutathione peroxidase 6, mitochondrial	99%
20	NW_021638508	pathogensis-related protein CP26	99%
21	NC_041792	chlorophyll a-b binding protein CP26, chloroplastic	99%
22	NC_041793	chlorophyll a-b binding protein of LHCII type 1-like	99%
23	NC_041803	F-box protein AFR-like	99%

## Data Availability

Not applicable.

## Supplementary Materials

The following supporting information can be downloaded at: www.mdpi.com/article/10.3390/ijms23042033/s1.
