# Supplementary material for: MdSCL8 as a Negative Regulator Participates in ALA-Induced FLS1 to Promote Flavonol Accumulation in Apples
Source: Int J Mol Sci. 2022 Feb 12;23(4):2033. doi: 10.3390/ijms23042033 (PMC8875840; doi:10.3390/ijms23042033)
Supplement: Supplementary file 1 [file ijms-23-02033-s001.zip › ijms-1566756-supplementary.pdf]

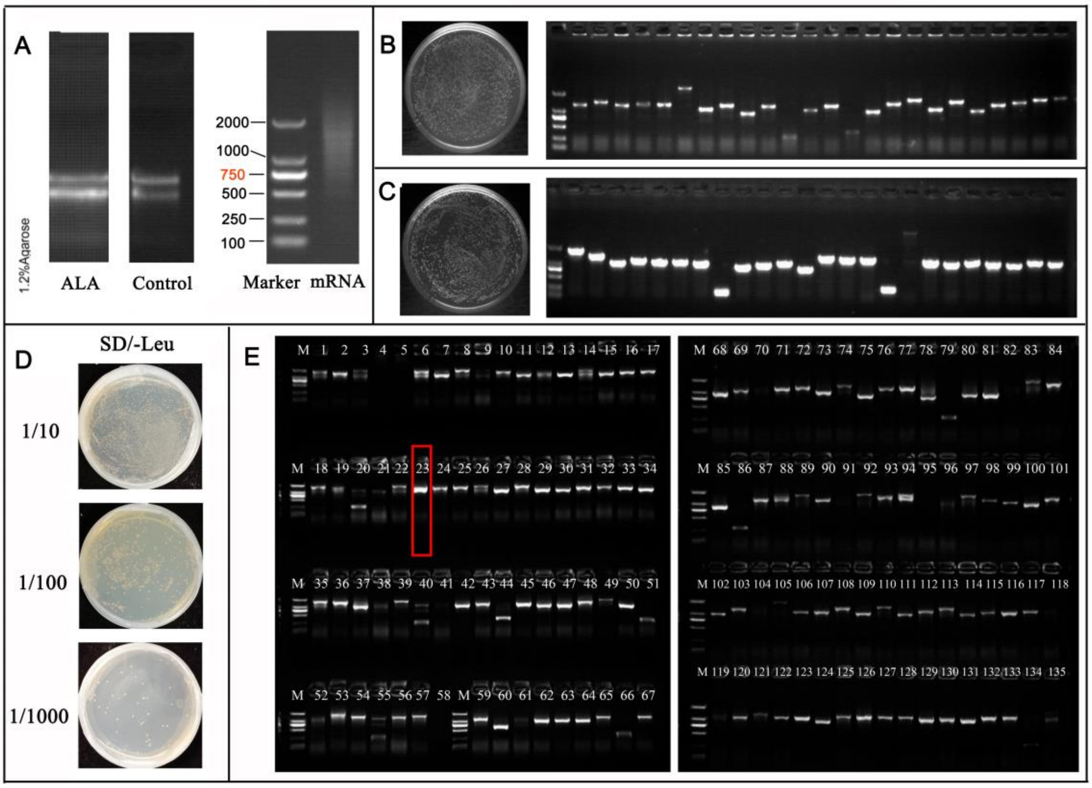

Figure S1. Construction of apple calli cDNA library, screening of yeast one-hybrid cDNA library and PCR detection of positive colonies. (A) Agarose gel electrophoresis analysis of total RNA and mRNA extracted from apple calli. Total RNA electrophoresis map, marker, mRNA electrophoresis map. (B) Identification of cDNA primary library capacity and agarose gel electrophoresis analysis of inserted fragment sizes. (C) Identification of cDNA secondary library capacity and agarose gel electrophoresis analysis of inserted fragment size. (D) The screening of yeast one-hybrid cDNA library. 1/10, 1/100, and 1/1000 respectively represent 10 times, 100 times, and 1000 times diluted conversion. (E) Yeast colony PCR detection of yeast from figure D. MdSCL8 is marked with red rectangle.

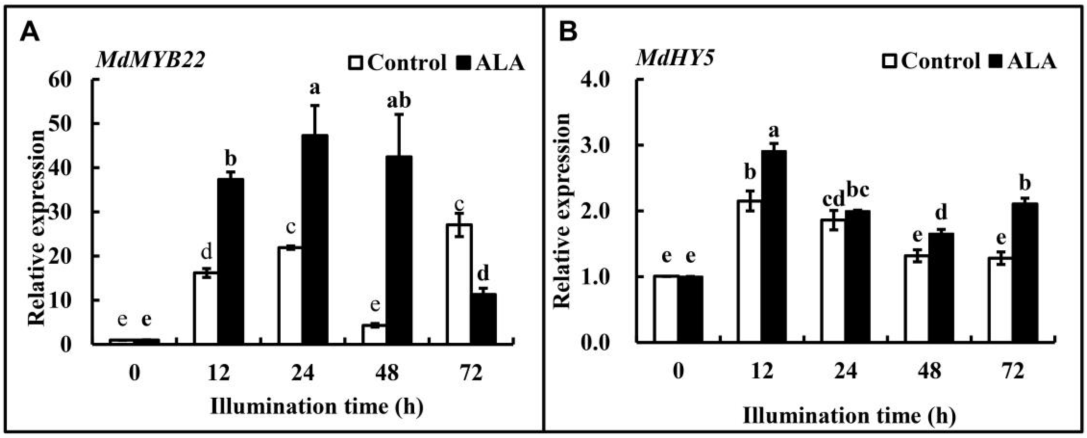

Figure S2. Effect of ALA on *MdMYB22* and *MdHY5* gene expression in calli. The calli were treated with 50 mg L^{-1} ALA or deionized water (Control) for 3 h under dark and then cultured in solid MS medium under light of 100 μmol $m^{-2}s^{-1}$ photon flux density at 22℃ for 72 h. The values of (A, B) were performed 3 biological replicates. Error bars represent standard errors. Three biological replicates were performed for each experiment. The same lowercase letters indicate no significant differences at $P \leq 0.05$.

Table S1. Primer sequences used in the study

	Primer name	Froward primer5'-3'	Reverse primer5'-3'
CDS amplification	*121-SCL8*	GAGAACACGGGGGACTCTAGAATG CAATCGGGAGGCTTCAACG	GGACTGACCACCCGGGGATCCACG CCAAGCGGAAGCGAC
	1300-SCL8	GAGAACACGGGGGACTCTAGAATG CAATCGGGAGGCTTC	GCCCTTGCTCACCATGGATCCACGC CAAGCGGAAGCGAC
	0800-MdFLS1	TCGACGGTATCGATAAGCTTTAGGT GTGAAATTCTGTCTTTAGGACA	GCTCTAGAACTAGTGGATCCTTCCA CCGCTCTCATCTCTCTT
	proMdFLS1	GAGAACACGGGGGACTCTAGATAG GTGTGAAATTCTGTCTTTAGG	GGACTGACCACCCGGGGATCCTTCC ACCGCTCTCATCTCTC
	MdSCL8(i)	GGGGACAAGTTTGTACAAAAAAGC AGGCTCCCACCAGCAGAACCACTAC	GGGGACCACTTTGTACAAGAAAGCT GGGTGCTCTTGCAGCGTGTTACTGA
	MdHY5(i)	GGGGACAAGTTTGTACAAAAAAGC AGGCTTCTTCGAGCTCTGCATTCCA	GGGGACCACTTTGTACAAGAAAGCT GGGTAACAACCTCTTCAGCCGCTT
	MdMYB22(i)	GGGGACAAGTTTGTACAAAAAAGC AGGCTTGAAATTAGCACCAGTGTCC	GGGGACCACTTTGTACAAGAAAGCT GGGTGGGACCTCCATTAGTAGCACG
qRT-PCR	*MdUBQ*	CTCCGTGGTGGTTTTTAAGT	GGAGGCAGAAACAGTACCAT
	MdFLS1	AAAATGGGAGTGGAGTCTGTG	TTATGACCTCGCTGGGAATGT
	MdSCL8	CGTGGAAAGATGTGAAGTGTT	GCGGCTTATCCAACCAAA
	MdHY5	GGAAGAGTGCCGGAAATCG	CTCCCTTGCTTGCTGTGCT
	MdMYB22	GTAATAGGTGGTCTTTGATAGCG	GGGTTTGGTGGTAGAGTAGGT
Y2H/ Y1H	*BD-MdHY5*	ATGGCCATGGAGGCCGAATTCATGC AAGAGCAGGCGACG	CCGCTGCAGGTCGACGGATCCATCC GCATTTGCACCACCA
	AD-MdMYB22	GCCATGGAGGCCAGTGAATTCATGG GGAGGGCGCCGTGC	CAGCTCGAGCTCGATGGATCCCTGG ATGCCGTAGTAAGTCGTCG
	AD-MdHY5	GCCATGGAGGCCAGTGAATTCATGC AAGAGCAGGCGACG	CAGCTCGAGCTCGATGGATCCATCC GCATTTGCACCACCA
	BD-MdSCL8	ATGGCCATGGAGGCCGAATTCATGC AATCGGGAGGCTTCA	CCGCTGCAGGTCGACGGATCCACGC CAAGCGGAAGCGAC
	AD-MdSCL8	GTGGGCATCGATACGGGATCCATGC AATCGGGAGGCTTCA	ACGATTCATCTGCAGCTCGAGACGC CAAGCGGAAGCGAC
	proMdFLS-pAbAi	AAATGATGAATTGAAAAGCTTTAGG TGTGAAATTCTGTCTTTAGGACA	ATACAGAGCACATGCCTCGAGTTCC ACCGCTCTCATCTCTCTT
BIFC	*YNE-MdHY5*	GAGAACACGGGGGACTCTAGAATG CAAGAGCAGGCGACG	CTCCATCCCGGGAGCGGTACCATCC GCATTTGCACCACCA
	YNE-MdSCL8	GAGAACACGGGGGACTCTAGAATG CAATCGGGAGGCTTCA	CTCCATCCCGGGAGCGGTACCACGC CAAGCGGAAGCGAC

	YCE-MdMYB22	GAGAACACGGGGGACTCTAGAATGGGGAGGGCGCCGTGC	GTACATCCCGGGAGCGGTACCCTGGATGCCGTAGTAAGTCGTCG
	YCE-MdSCL8	GAGAACACGGGGGACTCTAGAATGCAATCGGGAGGCTTCA	GTACATCCCGGGAGCGGTACCACGCCAAGCGGAAGCGAC
Luciferase	*NLUC-MdHY5*	GAGCTCGGTACCCGGGGATCCATGCAAGAGCAGGCGACG	CGCGTACGAGATCTGGTCGACATCCGCATTTGCACCACCA
	CLUC-MdMYB22	TACGCGTCCCGGGGCGGTACCATGGGGAGGGCGCCGTGC	TGTAGTCCATTTGTTGGATCCCTGGATGCCGTAGTAAGTCGTCG
	NLUC-MdSCL8	GAGCTCGGTACCCGGGGATCCATGCAATCGGGAGGCTTCA	CGCGTACGAGATCTGGTCGACACGCCAAGCGGAAGCGAC
	CLUC-MdSCL8	TACGCGTCCCGGGGCGGTACCATGCAATCGGGAGGCTTCA	TGTAGTCCATTTGTTGGATCCCGCCAAGCGGAAGCGAC
